# Community Knowledge About Climate Change and Industrialization Impacts on Recurrence of Dengue Epidemics in Selected Districts in Tanzania: A Cross‐Sectional Study

**DOI:** 10.1002/hsr2.70745

**Published:** 2025-04-28

**Authors:** Clement N. Mweya, Simeon P. Mwanyonga, Liness A. Ndelwa, Joyce Massaro

**Affiliations:** ^1^ Mbeya College of Health and Allied Sciences University of Dar es Salaam Mbeya Tanzania; ^2^ Mbeya Medical Research Center National Institute for Medical Research Mbeya Tanzania; ^3^ Department of Psychiatry and Mental Health Mbeya Zonal Referral Hospital Mbeya Tanzania; ^4^ Department of Pediatrics and Child Health Mbeya Zonal Referral Hospital Mbeya Tanzania

**Keywords:** climate change, community knowledge, dengue, industrialization, Tanzania

## Abstract

**Background and Aims:**

Dengue fever epidemics pose an increasing public health threat in Tanzania. Climate change and industrialization may influence outbreaks, while community knowledge plays a vital role in prevention. This study examined public knowledge about environmental and anthropogenic impacts on dengue transmission.

**Methods:**

A cross‐sectional study was conducted from April to June 2022 with 482 participants from Bahi, Kyela, and Ngorongoro districts. A validated questionnaire assessed demographic characteristics and knowledge about dengue epidemiology related to climate and industrialization. Multinomial logistic regression and *χ* ^2^ tests examined associations between variables.

**Results:**

Over half of the participants (52.9%) were male, and most were aged 26–35 (33.2%). Only 21% demonstrated a good understanding of industrialization's health impacts, while 19% knew the climate change linkage with dengue. Significant knowledge gaps exist regarding climate change and industrialization impacts linked to recurrent epidemics (44.2% poor knowledge). Age over 35 (AOR 1.73, 95% CI 1.39–2.14), primary education or less (AOR 0.77, 95% CI 0.59–0.99), and unemployment (AOR 0.31, 95% CI 0.23–0.42) were associated with poor knowledge. Gender and occupation significantly predicted climate change knowledge (*p* < 0.001).

**Conclusion:**

Communities in dengue‐endemic areas have limited knowledge about climate and anthropogenic drivers of recurring epidemics. Targeted educational interventions can improve understanding and preventative behavior among high‐risk demographics.

## 1 Introduction

Dengue fever remains a significant public health threat in Tanzania and globally, with recurring epidemics alarming public health authorities [1]. Dengue fever likely originated in Africa or Asia and spread globally through the slave trade and colonization in the 17th and 18th centuries [2, 3]. Major epidemics arose in the late 18th century, including outbreaks reported in Africa, North America, Spain, Greece, and Australia [4]. In the 20th century, dengue epidemics increased as urbanization and travel expanded, with outbreaks in Greece, Taiwan, the South Pacific, Cuba, and more [5]. After World War II, epidemics accelerated in Southeast Asia. The 1950s–60s saw major outbreaks in the Philippines and Thailand [2]. Dengue fever has been endemic in Tanzania for over 50 years, with periodic outbreaks. The first confirmed epidemic was reported in 2010 in Dar es Salaam. Major epidemics occurred in 2014 and 2019, primarily affecting Dar es Salaam, Tanga, Mbeya, Mwanza, and other regions. The 2019 dengue outbreak was the largest on record, with over 11,000 cases and seven deaths. Strains DEN‐2 and DEN‐3 were confirmed. Smaller outbreaks continued in 2020–2021 in Dar es Salaam, Tanga, Mwanza, and Zanzibar [6]. Over 2500 cases were reported during this period [1].

Mounting evidence indicates that climate change and industrialization may contribute to the disease's resurgence [7, 8]. In combating dengue, community knowledge is crucial for preventing its spread. While industrialization has long been associated with adverse health risks contrasting with its economic benefits, the relationship between climate and health has generally been considered more positive [9]. However, changes in temperature, precipitation, and climate variability can impact human health mainly through influences on disease transmission and food production [10]. Climate change is deemed a global threat for emerging vector‐borne disease risk areas. Projections for Tanzania indicate a potential temperature increase of 1°C–3°C above baseline averages by the 2050s, increased rainfall, and more extreme flooding and drought events [11]. Climate may alter suitable conditions for mosquito vectors that transmit and sustain Rift Valley fever virus and dengue in Tanzania [12, 13]. Rising temperatures and more significant climate variability threaten to undermine recent global progress against these diseases [14].

The relationship between industrialization, climate change, and infectious diseases is complex and multifaceted. Industrialization can lead to increased greenhouse gas emissions, contributing to global warming and climate change. These changes can alter ecosystem dynamics, potentially expanding the geographical range of disease vectors like mosquitoes. In Tanzania, rapid industrialization has been observed in urban centers like Dar es Salaam, potentially altering local microclimates and creating new breeding grounds for mosquitoes. Climate change, characterized by rising temperatures and changing precipitation patterns, can further exacerbate these effects by extending the breeding season of mosquitoes and accelerating their life cycles. Understanding these mechanisms is crucial for developing effective strategies to mitigate the impact of dengue epidemics in the face of ongoing industrialization and climate change.

Little data exist regarding community knowledge about dengue fever risk factors and transmission patterns in Tanzania. Enhanced public understanding of how climate change and industrialization impact dengue epidemiology could promote preventative behavior, strengthen disease surveillance, and improve outbreak response. This study seeks to evaluate gaps in community knowledge through a cross‐sectional survey in a region of Tanzania affected by repeated dengue epidemics. Assessing knowledge gaps related to disease vectors, transmission, and their relationship with climate and industry can illuminate areas needing targeted education. In this study, we defined the knowledge gap as a significant discrepancy between the current understanding of a topic within a community and the level of understanding necessary for effective disease prevention and control. Specifically, we consider a knowledge gap to exist when < 50% of the surveyed population demonstrates a good understanding of a particular aspect of dengue epidemiology, climate change impacts, or industrialization effects. Our findings will guide public health initiatives to increase community education and engagement surrounding dengue prevention and control strategies. Effective interventions must improve public understanding of dengue fever epidemiology while considering local cultural and social contexts. Ultimately, community knowledge and involvement in surveillance and vector control are essential to mitigate large‐scale dengue outbreaks exacerbated by climate change and industrialization.

## 2 Materials and Methods

### 2.1 Study Area

**Figure 1:**
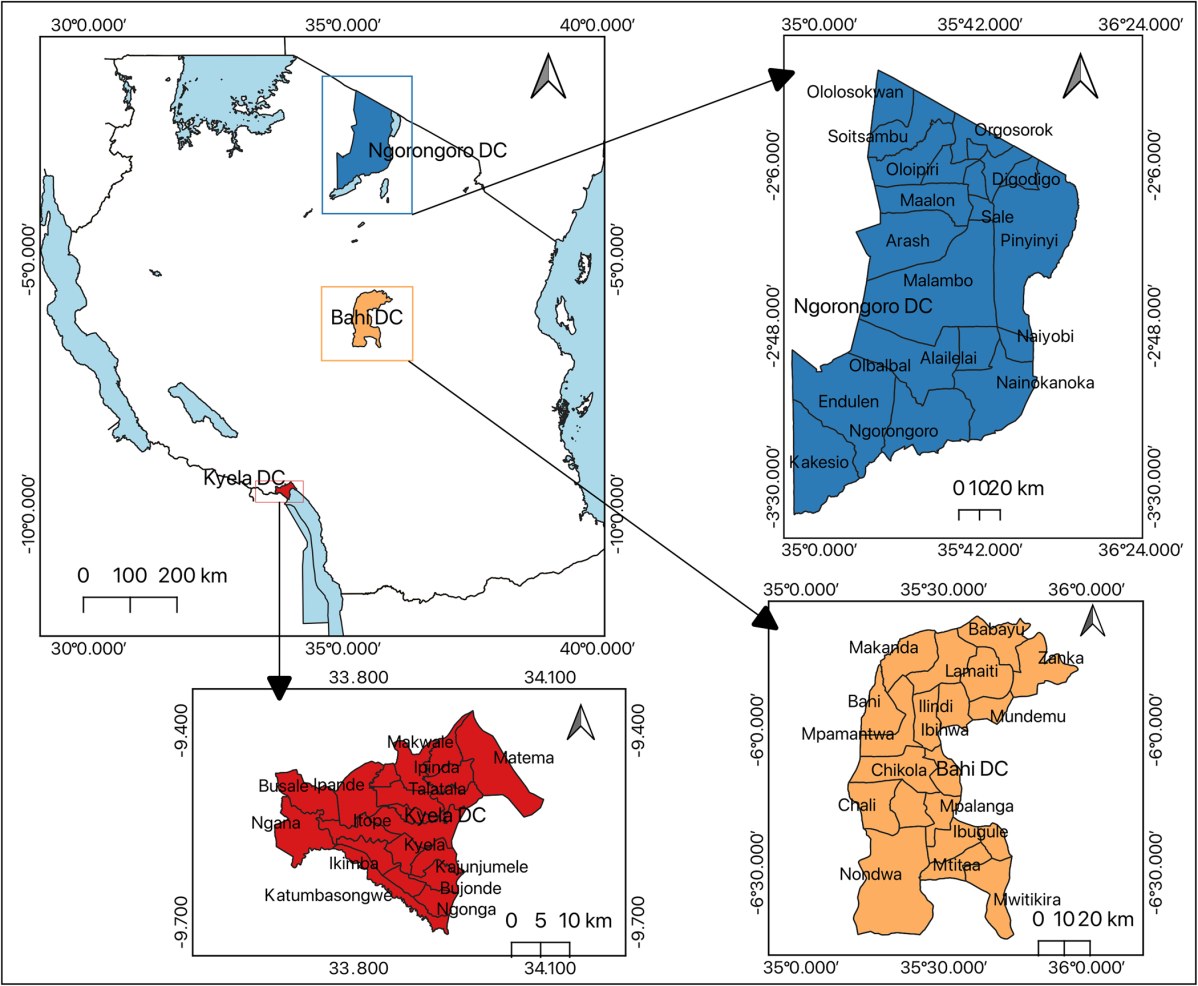
Map indicating study areas in Ngorongoro, Bahi, and Kyela districts.

Recognizing the vital role of local communities in preventing dengue spread, this descriptive cross‐sectional survey was conducted among residents across selected villages in the Kyela, Bahi, and Ngorongoro districts of Tanzania. Kyela, situated in the Great Rift Valley flood plains of Lake Nyasa, was chosen due to evidence of interepizootic arbovirus circulation, including Rift Valley fever, among domestic animals [15]. Located in Tanzania's central zone with a semi‐arid climate, Bahi suffered greatly during the 2006–2007 Rift Valley fever outbreak and exhibited viral antibody persistence in cattle [16, 17]. Ngorongoro, renowned for unique human–livestock–wildlife interactions within the Serengeti‐Masai Mara ecosystem, has a history of arbovirus epidemics like Rift Valley fever [18]. Engaging these diverse communities offers crucial perspectives on localized knowledge gaps, behaviors, and needs to inform targeted dengue prevention strategies and educational interventions (Figure 1).

### 2.2 Study Design and Recruitment of Participants

This descriptive, community‐based, cross‐sectional survey was conducted from April to June 2022. The study engaged participants from the Kyela, Ngorongoro, and Bahi districts. The study population consisted of a statistically representative sample of participants 18 years and above. A sample size of 482 participants was utilized in this study, determined by calculating the minimum required sample size and adjusted based on the total combined population of 617,413 across the Bahi, Kyela, and Ngorongoro districts [19, 20]. A multistage random selection process was utilized to identify participating villages within the specified districts. A simple random sampling was then employed to select individual participants from the chosen villages.

### 2.3 Questionnaire Development and Validation

The structured questionnaire used in this study was developed based on a comprehensive literature review and consultation with experts in epidemiology, climate science, and public health. The questionnaire covered two main domains: (1) understanding of climate change impacts on health and (2) industrialization effects on disease patterns. The questionnaire was initially piloted with a sample of 50 individuals from a nonstudy area to assess clarity and comprehensibility. Based on the pilot results, the necessary modifications were made. The internal consistency of the final questionnaire was assessed using Cronbach's *α*, yielding a value of 0.82, indicating good reliability [21] Knowledge levels were categorized as follows: No knowledge: 0%–30% correct answers; Fair knowledge: 31%–70% correct answers; and Good knowledge: 71%–100% correct answers.

### 2.4 Inclusion and Exclusion Criteria

Inclusion criteria for participants included age being 18 years or older, resident of the study area for at least 1 year and the ability to provide informed consent. Exclusion criteria were the inability to communicate effectively in Swahili or English and severe cognitive impairment, which would prevent understanding the questionnaire.

### 2.5 Data Collection

Structured questionnaires were administered through in‐person interviews to assess knowledge regarding climate change, industrialization, and dengue epidemics in Tanzania. The survey instrument incorporated validated procedures from previous studies with refinements where needed [22, 23]. Interviewers guided participants and clarified key points about the questionnaire responses. Data collected encompassed demographics, understanding of dengue outbreaks, and perspectives on how industrialization influences epidemics and climate change interactions. The questionnaire assessed knowledge about dengue and industrialization, industrialization's influence on climate change and the linkage between climate change and industrialization with dengue epidemic recurrence. Participants were assured of confidentiality, with all information obtained used strictly for research purposes.

### 2.6 Statistical Analysis

Data collected was stored in Excel spreadsheets and then imported into IBM SPSS Statistics 23.0 for analysis. Descriptive and inferential statistics were used to characterize the data, following standard epidemiological methods. Categorical variables were summarized with counts and percentages. *χ* ^2^ tests evaluated potential associations between sociodemographics and poor (no knowledge), fair or good levels of dengue knowledge as dependent variables. Multinomial logistic regression additionally assessed predictors of knowledge outcomes. All analyses were prespecified in our study protocol. We did not conduct any exploratory or subgroup analyses beyond those originally planned. The significance level was set at *α* = 0.05 for all statistical tests. All tests were two‐sided. Results with *p* < 0.05 were considered statistically significant. The analysis outputs were presented through text descriptions, figures, and tables to facilitate interpretation.

### 2.7 Ethical Approval

Ethical approval for this study was obtained from the National Institute for Medical Research's Medical Research Coordinating Committee under the project “Exploring Recurrence of Dengue Epidemics in Tanzania: A Climate Change and Industrialization Perspective” (Approval Number NIMR/HQ/R.8a/Vol. IX/3870). Additional permission to conduct research was requested from the Regional and District authorities. Verbal informed consent was secured from all willing participants to uphold ethical standards. Respondent confidentiality and privacy were paramount throughout the study. No names were recorded on data collection instruments, further maintaining anonymity.

## 3 Results

### 3.1 Sociodemographic Characteristics of Study Participants by Districts (*n* = 482)

A total of 482 study participants from three districts in Tanzania—Bahi, Kyela, and Ngorongoro—were involved in the study. The majority of participants were male (52.9%), and most were aged 26–35 years (33.2%). Over one‐third had primary education (36.1%), while 17.4% had college certificates/diplomas, and only 4.4% had university degrees. More than half (57.3%) were unemployed, while 26.8% were self‐employed, 13.9% were informally employed, and just 2% were formally employed. The three districts had similar distributions of gender, age groups, education levels, and occupations among their participants, with each district contributing between 32.4% and 34% of the total participants (Table 1).

**Table 1:** Sociodemographic characteristics of study participants by districts (*n* = 482).

	Bahi	Kyela	Ngorongoro	
Gender				
Male	86 (17.8%)	88 (18.3%)	81 (16.8%)	255 (52.9%)
Female	78 (16.2%)	74 (15.3%)	75 (15.6%)	227 (47.1%)
	164 (34.0%)	162 (33.6%)	156 (32.4%)	482
Age group				
18–25	32 (6.6%)	39 (8.1%)	24 (5.0%)	95 (19.7%)
26–35	48 (10.0%)	57 (11.8%)	55 (11.4%)	160 (33.2%)
36–45	32 (6.6%)	32 (6.6%)	30 (6.2%)	94 (19.5%)
46–55	24 (5.0%)	22 (4.6%)	27 (5.6%)	73 (15.1%)
≥ 56	28 (5.8%)	12 (2.5%)	20 (4.2%)	60 (12.5%)
	164 (34.0%)	162 (33.6%)	156 (32.4%)	482
Level of education				
No formal education	24 (5.0%)	12 (2.5%)	21 (4.3%)	57 (11.8%)
Primary education	44 (9.1%)	70 (14.5%)	60 (12.5%)	174 (36.1%)
Secondary education	48 (10.0%)	56 (11.6%)	42 (8.7%)	146 (30.3%)
College (diploma/certificate)	36 (7.4%)	21 (4.4%)	27 (5.6%)	84 (17.4%)
University (degree)	12 (2.5%)	3 (0.6%)	6 (1.3%)	21 (4.4%)
	164 (34.0%)	162 (33.6%)	156 (32.4%)	482
Occupation				
Formally employed	4 (0.8%)	3 (0.6%)	3 (0.6%)	10 (2.0%)
Informally employed	20 (4.1%)	26 (5.4%)	21 (4.4%)	67 (13.9%)
Self‐employed	48 (10.0%)	39 (8.1%)	42 (8.7%)	129 (26.8%)
Unemployed	92 (19.1%)	94 (19.5%)	90 (18.7%)	276 (57.3%)
	164 (34.0%)	162 (33.6%)	156 (32.4%)	482

### 3.2 Knowledge About Dengue, Industrialization, and Climate Change

#### 3.2.1 Dengue and Industrialization

Most participants (47%) had a fair knowledge about industrialization. Ngorongoro district contributed the most to fair knowledge (16%), followed by Bahi (15%) and Kyela (15%). There was poor knowledge in 156 (32%), while 102 (21%) had good knowledge about industrialization. More females (22%) had fair knowledge and more (21%) had poor knowledge. Males showed higher good knowledge (18%) than females (3%) (*χ* ^2^ = 62.93, *p* < 0.001). Those aged 26–35 showed the highest fair (25%) and good knowledge (8%). Age group, 36–45 showed the highest poor knowledge (13%) (*χ* ^2^ = 155.73, *p* < 0.001). Good knowledge was highest among those with secondary education (10%), while fair and poor knowledge were highest among those with primary education (18% and 13%, respectively) (*χ* ^2^ = 68.20, *p* < 0.001). Self‐employed people had the highest level of good knowledge (16%). Unemployed had the highest fair (28%) and poor knowledge (27%) (*χ* ^2^ = 200.06, *p* < 0.001). Females had 88% lower odds of higher knowledge (AOR 0.12; 95% CI 0.06–0.25) than males, alongside those over 35 with 39%–73%. Lesser educated groups were marginally associated with poorer comprehension (AOR 1.35; 95% CI 0.98–1.87). Unemployed participants demonstrated substantially lower good understanding versus employed counterparts (AOR 0.16; 95% CI 0.10–0.24) (Table 2).

**Table 2:** Association between sociodemographic characteristics and knowledge about dengue and industrialization, climate change, and dengue epidemics.

Characteristic	No knowledge	Fair knowledge	Good knowledge
	AOR (95% CI)	AOR (95% CI)	AOR (95% CI)
Dengue and industrialization			
Gender (female vs. male)	0.12 (0.06–0.25)	0.26 (0.14–0.51)	1.00 (Ref.)
Age (> 35 vs. ≤ 35 years)	1.39 (1.07–1.82)	0.86 (0.68–1.08)	1.00 (Ref.)
Level of education (< university)	1.35 (0.98–1.87)	1.08 (0.82–1.44)	1.00 (Ref.)
Occupation (unemployed vs. employed)	0.16 (0.10–0.24)	0.31 (0.22–0.42)	1.00 (Ref.)
Industrialization's influence on climate change			
Gender (female vs. male)	0.07 (0.03–0.16)	0.11 (0.05–0.23)	1.00 (Ref.)
Age (> 35 vs. ≤ 35 years)	1.78 (1.37–2.32)	1.44 (1.14–1.81)	1.00 (Ref.)
Level of education (< university)	1.48 (1.08–2.02)	0.96 (0.72–1.26)	1.00 (Ref.)
Occupation (unemployed vs. employed)	0.25 (0.17–0.37)	0.82 (0.61–1.10)	1.00 (Ref.)
Climate change and industrialization and dengue epidemic recurrence			
Gender (female vs. male)	0.40 (0.24–0.66)	0.73 (0.42–1.26)	1.00 (Ref.)
Age (> 35 vs. ≤ 35 years)	1.73 (1.39–2.14)	0.84 (0.67–1.07)	1.00 (Ref.)
Level of education (< university)	0.77 (0.59–0.99)	0.54 (0.41–0.72)	1.00 (Ref.)
Occupation (unemployed vs. employed)	0.31 (0.23–0.42)	0.70 (0.53–0.94)	1.00 (Ref.)

*Note:* Reference categories are male for gender, ≤ 35 years for age, university degree for level of education, and employed for occupation.

Abbreviations: AOR, adjusted odd ratios; CI, confidence interval; Ref, reference category.

#### 3.2.2 Industrialization's Influence on Climate Change

**Figure 2:**
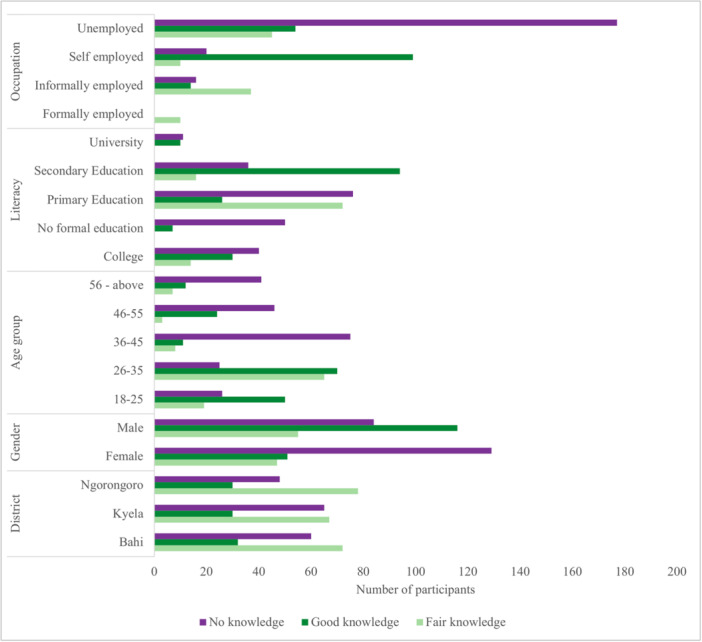
Knowledge of study participants on industrialization's influence on climate change.

Most participants, 482 (45%), knew about industrialization's influence on climate change fairly well. Ngorongoro district contributed the most to fair knowledge (16.2%), followed by Bahi (14.9%) and Kyela (13.9%). Poor knowledge was observed in 173 participants (36%). Only 92 (19%) had good knowledge, evenly distributed across districts. More males (17%) than females (2%) had good knowledge, while more females (27%) had poor knowledge (*χ* ^2^ = 33.92, *p* < 0.001). Those aged 26–35 showed the highest good knowledge (16%), while ages 36–45 showed the highest poor knowledge (14%) (*χ* ^2^ = 155.51, *p* < 0.001). Good knowledge was highest among those with secondary education (9%), and poor knowledge was the highest among those with primary education (16%) (*χ* ^2^ = 161.74, *p* < 0.001). Self‐employed participants showed the highest level of good knowledge (7%). Unemployed participants showed the highest fair (22%) and poor knowledge (31%) (*χ* ^2^ = 230.90, *p* < 0.001) (Figure 2). Knowledge about industrialization's climate impacts showed similar disparities, with females exhibiting 93% decreased odds compared to males (AOR 0.07; 95% CI 0.03–0.16). Ages over 35 years, lower education levels and unemployment status also carried a 44%–78% reduced climate literacy. At the moderate knowledge level, age and education distinctions diminished. Gender and occupation status significantly predicted climate change comprehension (*p* < 0.001) (Table 2).

#### 3.2.3 Climate Change and Industrialization and Dengue Epidemic Recurrence

**Figure 3:**
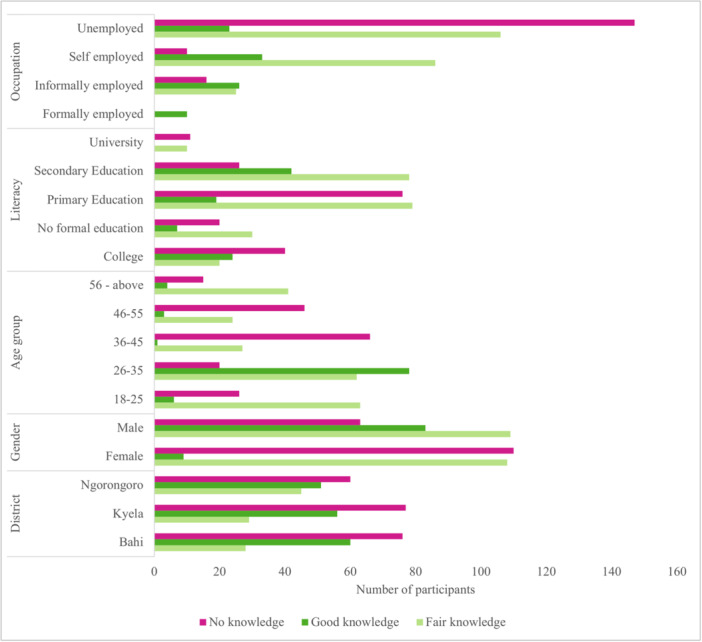
Knowledge of study participants on climate change and industrialization and recurrence of dengue epidemics.

Of the 482 participants, 213 (44%) had poor knowledge of the link between climate change, industrialization, and dengue epidemic recurrence. Kyela district contributed the most to poor knowledge (16%), followed by Bahi (15.8%) and Ngorongoro (12.4%). There was good knowledge in 167 participants (35%), while 102 (21%) had moderate knowledge. More females (27%) than males (24%) had poor knowledge, whereas more males had good knowledge. There was a significant association between gender and knowledge (*χ* ^2^ = 70.91, *p* < 0.001). Those aged 26–35 showed the highest good knowledge (15%), while ages 36–45 showed the highest poor knowledge (16%) (*χ* ^2^ = 211.56, *p* < 0.001). Good knowledge was highest among those with secondary education (20%), and poor knowledge was the highest among those with primary education (16%) (*χ* ^2^ = 55.12, *p* < 0.001). Self‐employed participants showed the most suitable knowledge (21%), while the unemployed showed the most poor knowledge (37%) (*χ* ^2^ = 146.96, *p* < 0.001) (Figure 3). Knowledge connecting climatic and industrial changes with recurrent dengue epidemics was weakest among groups over 36 years old, with 56% lower knowledge than youth (AOR 0.44; 95% CI 0.30–0.64). While gender carried a 40% gap (AOR 0.6; 95% CI 0.36–0.99) in higher knowledge, this disparity disappeared for moderate comprehension. Unemployed individuals knew 31% less than their employed counterparts (AOR 0.69; 0.51–0.93). All characteristics significantly predicted no knowledge levels (*p* < 0.001) (Table 2).

## 4 Discussion

This study found gaps in knowledge about industrialization's impacts, with less than half of the participants demonstrating a good understanding. Similar knowledge gaps related to climate change and health have been reported in other contexts in developing countries. A study in rural India found a limited understanding of the health impacts of climate change, with less education being associated with lower knowledge [24, 25]. Researchers in Nigeria reported poor knowledge of climate change among university students, highlighting the need for improved climate education [26]. Our findings align with these studies showing knowledge gaps, especially among those with less education. Lower climate change and industrialization knowledge among women versus men also mirrors the gender divides seen elsewhere. A study in Bangladesh found that women had significantly lower climate change knowledge than men [25]. Social and cultural gender norms may limit women's access to information sources. Targeting educational interventions toward lower education groups and women could help improve community understanding of climate–health linkages in this region.

Our findings reveal significant knowledge gaps regarding the links between climate change, industrialization, and dengue transmission. We define a “good understanding” as correctly answering at least 71% of the questions related to a specific topic. Using this criterion, only 21% of participants demonstrated a good understanding of industrialization's health impacts, while a mere 19% showed good knowledge about the climate change connections with dengue. These results indicate a critical need for targeted educational interventions to bridge these knowledge gaps (Table 2 and Figure 2).

Industrialization has generally been thought to have a more favorable link with human health, despite urbanization and trade's long‐standing adverse health hazards [9]. However, our findings indicate that of the participants, 46.5% had only fair knowledge connecting industrialization's role in dengue epidemics. While industrialization brings medical advancements, improved food supplies, and higher living standards [9], 45% still only had a fair knowledge of its influence on climate change. Climate change severely impacts environmental degradation, whereas industrialization has a positive environmental impact [27]. Men demonstrated better knowledge of climate change than women, possibly because men were more concerned with crops and livestock while women focused on household food availability [28]. Generally, participants failed to link industrialization with its impacts on dengue despite some knowledge of climate change impacts.

This study found significant knowledge gaps regarding industrialization's influence on climate change, with less than one‐fifth of participants demonstrating a good understanding. These findings align with other research from developing regions showing limited public comprehension of climate change drivers and impacts. A study in India found poor knowledge of the link between industrialization, greenhouse gas emissions, and climate change, particularly among less educated groups [29]. Researchers in Kenya reported low knowledge of climate change causes among rural communities, attributed partly to minimal exposure to education [30]. As in the current study, men and those with more education showed greater climate change knowledge. The considerable knowledge gaps identified here underscore the need for improved climate education and communication campaigns tailored to this context. Women and less educated population segments should be prioritized to bridge gender and socioeconomic divides in climate literacy. Targeted outreach and training programs for specific subgroups have proven effective for building climate change knowledge in other developing country settings [31]. As climate change accelerates, enhancing community understanding of contributing factors like industrialization will be critical. Closing knowledge gaps can empower vulnerable groups to adapt and build resilience against climate change impacts.

Our findings regarding participants' knowledge of climate change and industrialization's role in dengue transmission and epidemics are noteworthy. In total, 44.2% of participants showed poor knowledge. Climate change is considered a significant factor in increasing dengue transmission intensity [32]. Similar studies in Tanzania show climate impacts suitability for mosquito breeding and dengue transmission [12, 13] due to rising temperatures. As participants know less about climate change and industrialization's significant health impacts on dengue epidemics, government action is needed to maximize educational activities in these communities. With limited studies predicting dengue based on climate and industrialization using machine learning [33], further work is warranted. Participants' poor knowledge highlights the need for increased education on climate and industrialization's links to dengue, given evidence that these factors influence vector breeding suitability and transmission. Targeted education could strengthen prevention and preparedness. More research applying machine learning to predict outbreaks based on climate and industrialization trends would also be beneficial.

The poor knowledge about climate change and industrialization's links to dengue transmission in this study echoes findings from similar research in dengue‐endemic areas. A study in Malaysia found limited public knowledge about the relationship between climate factors like temperature and rainfall with dengue incidence [34]. Researchers in Indonesia reported knowledge gaps in recognizing associations between environmental conditions, vector breeding sites, and dengue transmission [35]. As in the current study, climate‐dengue knowledge tended to be higher among those with more education. The significant gender discrepancies observed also mirror results from dengue studies elsewhere. A study in Pakistan found that women had much lower knowledge about dengue vectors, symptoms, and transmission compared to men [36]. Deeply rooted sociocultural gender norms likely contribute to differences in health knowledge and information access between genders. Overall, these findings highlight crucial gaps in community understanding of climate and environmental contributors to dengue epidemiology. Targeted outreach and education campaigns should focus on improving climate‐dengue literacy among women and less educated groups. Stronger knowledge can empower communities to implement preventive measures and seek timely care amidst climate change.

### 4.1 Current Programs and Interventions

In Tanzania, efforts to increase knowledge about industrialization and infectious diseases have been limited. The formal education system includes some coverage of climate change and environmental issues in secondary school curricula, but specific links to health outcomes are often not emphasized. The Ministry of Health has implemented sporadic public health campaigns on dengue prevention, but these have not consistently addressed the broader context of climate change and industrialization [37]. Nongovernmental organizations (NGOs) have conducted community‐based vector control programs, which include educational components [38]. However, these programs often focus on immediate prevention measures rather than long‐term environmental factors. Media coverage of dengue outbreaks has increased public knowledge to some extent, but in‐depth explanations of the underlying environmental and industrial factors are rare.

The private sector, including industries operating in dengue‐endemic areas, has shown limited engagement in corporate social responsibility (CSR) initiatives related to disease prevention. This represents a missed opportunity for collaboration between the public and private sectors in addressing this public health challenge [39]. Our findings underscore the need for more comprehensive, sustained efforts to educate the public about the complex relationships between industrialization, climate change, and infectious diseases like dengue. Future interventions should involve multiple stakeholders, including government agencies, educational institutions, NGOs, media outlets, and the private sector, to create a more informed and resilient population in the face of these evolving health threats.

### 4.2 Limitations

Our study limitations include: first, the sample size of 482 participants from only 3 districts, which may limit the generalizability of the findings. A larger, more representative national sample would provide greater insight. Second, the cross‐sectional design only assessed knowledge at a one‐time point; longitudinal monitoring could better evaluate knowledge trends. The exclusive focus on dengue fever also broadly provides a limited perspective on climate change knowledge. Finally, the inability to determine causality underscores the need for intervention studies demonstrating knowledge improvement over time. This study provides initial evidence of knowledge gaps, but the limitations highlight the need for more robust, expanded research on this topic in Tanzania and similar settings.

## 5 Conclusion

Our findings reveal critical knowledge gaps among dengue‐endemic communities regarding the environmental and industrial drivers of recurrent epidemics. Only 21% demonstrated a quality understanding of industrialization's health impacts, while merely 19% grasped climate change connections. Further, 44% showed inadequate knowledge of the links between these factors and disease patterns. Significant disparities emerged across demographics, with lower knowledge among women, older ages, lesser education levels and unemployed groups. These insights provide invaluable guidance for policymakers to implement targeted educational interventions and improve outbreak prevention through strategic public health initiatives. Introducing context‐specific teaching focused on high‐risk areas promises to increase community vigilance and reporting to mitigate large‐scale epidemics influenced by anthropogenic ecosystem alterations. Promoting climate literacy and community‐centered preparedness through specialized training offers a pathway to sustainable, locally driven solutions. Moving forward necessitates ongoing qualitative and quantitative research assessing the retention of information over time among priority subgroups. Nonetheless, facilitating participatory prevention via spaces for open dialog and exchanging cultural perspectives remains imperative for successful adoption. The urgency lies in actively engaging with communities to amplify expertise and provide appropriate tools to combat this growing menace, yielding dividends for future generations.

## Author Contributions

**Clement N. Mweya:** conceptualization, data curation, investigation, methodology, writing – review and editing, writing – original draft. **Simeon P. Mwanyonga:** conceptualization, data curation, investigation, writing – review and editing. **Liness A. Ndelwa:** conceptualization, data curation, investigation, writing – review and editing. **Joyce Massaro:** conceptualization, data curation, investigation, writing – review and editing.

## Ethics Statement

The protocol for this study was approved by the National Institute for Medical Research's Medical Research Coordinating Committee under the project “Exploring Recurrence of Dengue Epidemics in Tanzania: A Climate Change and Industrialization Perspective” (Approval Number NIMR/HQ/R.8a/Vol. IX/3870).

## Consent

Informed and written consent was obtained from all participants. The participant's confidentiality and anonymity were strictly maintained.

## Conflicts of Interest

The authors declare no conflicts of interest.

## Transparency Statement

The lead author Clement N. Mweya affirms that this manuscript is an honest, accurate, and transparent account of the study being reported; that no important aspects of the study have been omitted; and that any discrepancies from the study as planned (and, if relevant, registered) have been explained.

## Data Availability

The data used to support the findings of this study are available from the corresponding author upon request.
